# IGF-I and relation to growth in infancy and early childhood in very-low-birth-weight infants and term born infants

**DOI:** 10.1371/journal.pone.0171650

**Published:** 2017-02-09

**Authors:** Miranda de Jong, Anneke Cranendonk, Jos W. R. Twisk, Mirjam M. van Weissenbruch

**Affiliations:** 1 Department of Pediatrics, Albert Schweitzer Hospital, Dordrecht, The Netherlands; 2 Department of Pediatrics, Division of Neonatology, VU University Medical Center, Amsterdam, The Netherlands; 3 Department of Clinical Epidemiology and Biostatistics, VU University Medical Center, Amsterdam, The Netherlands; Garvan Institute of Medical Research, AUSTRALIA

## Abstract

**Background:**

In very-low-birth-weight infants IGF-I plays an important role in postnatal growth restriction and is probably also involved in growth restriction in childhood. We compared IGF-I and its relation to growth in early childhood in very-low-birth-weight infants and term appropriate for gestational age born infants.

**Methods:**

We included 41 very-low-birth-weight and 64 term infants. Anthropometry was performed at all visits to the outpatient clinic. IGF-I and insulin were measured in blood samples taken at 6 months and 2 years corrected age (very-low-birth-weight children) and at 3 months, 1 and 2 years (term children).

**Results:**

Over the first 2 years of life growth parameters are lower in very-low-birth-weight children compared to term children, but the difference in length decreases significantly. During the first 2 years of life IGF-I is higher in very-low-birth-weight children compared to term children. In both groups there is a significant relationship between IGF-I and (change in) length and weight over the first 2 years of life and between insulin and change in total body fat.

**Conclusions:**

Considering the relation of IGF-I to growth and the decrease in difference in length, higher IGF-I levels in very-low-birth-weight infants in early childhood probably have an important role in catch-up growth in length.

## Introduction

Insulin-like growth factor I (IGF-I) is important for fetal and postnatal growth and development. In contrast to term infants, IGF-I levels in preterm infants decrease after birth and only increase gradually during the early postnatal period [1]. Studies in preterm infants show that IGF-I levels during the first postnatal weeks are positively related to early postnatal growth [2–6]. IGF-I not only plays an important role in postnatal growth restriction; studies in older children (between 5 and 10 years of age) suggest that IGF-I also is involved in prolonged growth restriction in very-low-birth-weight (VLBW) infants [7, 8]. IGF-I levels in mid-childhood in preterm infants compared to term born children were found lower in one study [8], but higher in another study [9].

The aim of the present study was to compare IGF-I and its relation to growth parameters in infancy and early childhood in VLBW infants (birth weight < 1500 g) to term appropriate for gestational age (AGA) born infants.

## Methods

### Study population

The VLBW infants were part of the Neonatal Insulin Replacement Therapy in Europe (NIRTURE) trial, an international multicenter randomized controlled trial investigating the role of early insulin therapy in VLBW infants [10]. After written informed consent was obtained from both parents VLBW infants younger than 24 hours of age and requiring intensive care were randomized to receive continuous intravenous infusion of insulin for the first seven days of life or standard neonatal care with insulin treatment only in case of hyperglycemia. Exclusion criteria included maternal diabetes and major congenital anomalies. All infants participating in the NIRTURE trial in our neonatal intensive care unit (inclusion period from 2006 to 2007) were eligible for the present study. Therefore the sample size of the VLBW infants in the present study was determined by the number of infants we included in the NIRTURE trial.

The term infants were born between 2000 and 2005 from a low-risk population of pregnant women included in the first trimester in a prospective longitudinal study (Trophoblast study) which aimed to investigate the use of circulating trophoblast for prenatal diagnosis of pregnancy-associated diseases such as preeclampsia [11]. Only term infants born AGA were included in the present study. AGA was defined as a birth weight above the 10th percentile [12]. Standard deviation scores (SDS) of birth weight were calculated according to Niklasson [13].

Approval from the ethics committee of the VU University Medical Center was obtained.

During the inclusion period of the NIRTURE trial in our neonatal intensive care unit (21 months) 165 VLBW infants were admitted and the parents of 69 infants were approached regarding participation in the study. The most common reasons for not approaching parents were infants not requiring intensive care or no opportunity to obtain informed consent within the first 24 hours after birth. In our unit 47 VLBW infants participated in the NIRTURE trial. Five infants died and one child was excluded because parents refused blood sampling at the follow-up visits; 41 VLBW children were included in the present study. At 2 years corrected age one of these 41 children was lost to follow-up. Four (10%) of the VLBW children were SGA (defined as a birth weight below the 10th percentile [12]). Seventeen infants (9 male/8 female) were assigned to the early-insulin group and 24 infants (12 male/12 female) received standard neonatal care. During the first week of life 6 infants in the standard care group were treated with insulin for 1 or 2 days because of hyperglycemia due to sepsis.

90 term born infants were included in the follow-up part of the Trophoblast study of whom 72 were AGA. Eight AGA children were excluded from the present study because they were lost to follow-up after the first visit at 3 months of age; 64 children were included in the present study. At 2 years of age 6 children were lost to follow-up.

### Data collection

The VLBW infants visited the outpatient clinic at expected date of delivery and at the corrected ages of 3 and 6 months, 1 year and 2 years, the term born infants at 3 months, 1 year and 2 years of age, according to the protocol of the NIRTURE trial and Trophoblast study respectively. At each visit anthropometry according to Dauncey et al. [14] was performed by the same trained research nurse in all children. Body weight was measured using an electronic scale to the nearest 0.1 kg, standing height was measured to the nearest 0.1 cm and all lengths and circumferences were measured using a measuring tape to the nearest 0.1 cm. Body mass index (BMI) was calculated. Total body fat was calculated according to Dauncey from skinfold thickness measurements and body dimensions [14]. Standard deviation scores of weight, height, head circumference and BMI were calculated according to Dutch references [15, 16]. Blood samples for IGF-I and insulin measurement were taken at 6 months and 2 years corrected age in the VLBW infants and at 3 months, 1 and 2 years of age in the term born infants, according to the specific study protocol. All blood samples were taken early in the afternoon after a fasting period of at least 3 hours. Samples were stored at -80°C and were all analysed at the same time.

Study population and data collection also have been previously described [17–19].

### Assays

IGF-I in serum was measured by chemiluminescence immunoassay (Liaison, DiaSorin, Saluggia, Italy). Intra-assay coefficient of variation is 8% at both 10.3 nmol/l and 17.5 nmol/l and 9% at 23.8 nmol/l. Inter-assay coefficient of variation is 10% at 6.9 nmol/l, 7.4% at 30.8 nmol/l and 16% at 59.4 nmol/l.

Insulin in serum was measured by immunometric assay (Advia Centaur, Siemens Medical Solutions Diagnostics, Malvern, Pennsylvania). Lower limit of quantitation is 10 pmol/l. Intra-assay coefficient of variation is 4% at 20 pmol/l, 3% at 500 pmol/l and 4% at 1500 pmol/l. Inter-assay coefficient of variation is 8% at 24 pmol/l and 7% at both 780 pmol/l and 3000 pmol/l.

### Statistical analysis

Statistical analyses were performed using the Statistical Package of Social Sciences software for Microsoft Windows version 19 (SPSS inc., Chicago, Illinois) and Stata version 14 (StataCorp, College Station, Texas). Differences in characteristics between VLBW children and term AGA children were evaluated using Student's t-test for normally distributed values and Mann-Whitney test and Wilcoxon signed-rank test for not normally distributed values.

Longitudinal differences in anthropometry, IGF-I and insulin between the two groups were analysed with linear mixed model analyses. In a first analysis, growth parameters at different time-points were compared between the groups and by adding an interaction between group and time to the model it was evaluated whether the difference between the groups changed over time. In a second analysis, the differences in IGF-I and insulin between the groups on average over time were investigated. Because both IGF-I and insulin were highly skewed to the right, both variables were log transformed before analyses. Finally the longitudinal relationship between IGF-I and insulin on the one hand and growth parameters on the other hand was investigated. This was done for the absolute growth parameters as well as for the changes in growth parameters between subsequent time-points. Group differences in the observed relationships between IGF-I and insulin and growth parameters were investigated by adding an interaction between group and IGF-I and insulin to the linear mixed models. All analyses were (if possible) adjusted for gestational age, gender and target height.

For insulin levels below the limit of quantitation a value of 1 pmol/l was used. P values < 0.05 were considered as significant.

## Results

Table 1 shows the characteristics of the VLBW infants and term AGA infants including parental background information.

**Table 1:** Characteristics of the VLBW and term AGA children.

	VLBW (n = 41)	Term AGA (n = 64)	p-value
**Sex**	21 M / 20 F	35 M / 29 F	0.728
**Gestational age (wks)**	27.9 ± 1.3	39.3 ± 1.2	<0.001
**Birth weight (g)**	1059 ± 231	3529 ± 393	<0.001
**Birth weight SDS**	-0.06 ± 0.9	0.3 ± 0.7	0.019
**Maternal age (years)**	31.3 ± 4.7	33.7 ± 4.4	0.01
**Maternal weight (kg)**	68.2 ± 13.7	71.7 ±13.2	0.206
**Maternal smoking**	5/41 (12%)	6/64 (9%)	0.747
**Racial group**	26 Caucasian, 10 black, 3 Moroccan, 2 Asian	55 Caucasian, 4 black, 5 Asian	
**Highest level of parental education**^**a**^	3 low, 18 medium, 20 high	l 1ow, 19 medium, 35 high, 9 unknown	
**Target height SDS**	0.2 ± 0.9	0.5 ± 0.9	0.181
**Breast feeding**	31/41 (76%)	45/64 (70%)	0.554
- duration of exclusive breast feeding (months)	3 (0–8)	3 (0–6)	0.207
- total duration of breast feeding (months)	5 (1–23)	4 (1–24)	0.924
**Weight (g) at expected date of delivery**^**b**^	3154 ± 579		<0.001
**Weight SDS at expected date of delivery**^**b**^	-1.2 ± 1.3		<0.001

Data are expressed as mean ± standard deviation, percentages or numbers; duration of breast feeding is presented as median (range).

VLBW infants are compared to term AGA infants.

^a^Highest level of education completed by either parent was used as an indicator of socioeconomic status and classified as low (primary school, low occupational training), medium (high school, medium occupational training) or high (high occupational training, university).

^b^Weight (SDS) at expected date of delivery of the VLBW infants was compared to birth weight (sds) of the term AGA infants.

### Anthropometry

Table 2 shows the weight (SDS), length (SDS), head circumference (SDS), BMI (SDS) and total body fat at 3 months, 1 year and 2 years of (corrected) age for the VLBW and term AGA children. Longitudinal analysis showed that on average over the first 2 years of life all these growth parameters were significantly lower in VLBW children compared to term AGA children. The difference in length (SDS) between the VLBW children and term AGA children decreased significantly in the course of these 2 years. For the other growth parameters, the difference between the VLBW children and term AGA children increased or did not change over time.

**Table 2:** Anthropometry of the VLBW (n = 41) and term AGA (n = 64) children.

	VLBW	Term AGA	VLBW	Term AGA	VLBW	Term AGA	Difference^a^	p-value^a^	Interaction with time^b^
	*At 3 months (corrected) age*		*At 1 year (corrected) age*		*At 2 years (corrected) age*				
**Weight (kg)**	5.6 ± 0.9	6.2 ± 0.6	9.1 ± 1.2	10.1 ± 1.0	11.5 ± 1.3	13.0 ± 1.5	-1.3 (-1.9 to -0.8)	<0.001	negative
**Weight SDS**	-0.6 ± 1.4	0.5 ± 0.7	-0.9 ± 1.1	0.1 ± 0.8	-0.9 ± 1.0	0.1 ± 0.9	-1.0 (-1.3 to -0.7)	<0.001	no
**Length (cm)**	58.2 ± 2.7	61.4 ± 2.0	74.5 ± 2.9	76.5 ± 2.4	86.0 ± 3.7	87.7 ± 3.3	- 3.7 (-6.1 to -1.5)	0.001	positive
**Length SDS**	-1.1 ± 1.1	0.4 ± 0.7	-0.6 ± 1.0	0.1 ± 0.8	-0.7 ± 1.1	-0.3 ± 0.9	-0.9 (-1.2 to -0.5)	<0.001	positive
**Head circumference (cm)**	40.4 ± 1.5	41.3 ± 1.3	46.1 ± 1.6	47.2 ± 1.5	48.3 ± 1.7	49.4 ± 1.5	-1.3 (-2.1 to -0.6)	<0.001	no
**Head circumference SDS**	-0.1 ± 1.0	0.6 ± 0.8	-0.4 ± 0.9	0.4 ± 0.9	-0.2 ± 0.9	0.4 ± 0.9	-0.7 (-1.0 to -0.4)	<0.001	no
**BMI (kg/m2)**	16.3 ± 1.8	16.4 ± 1.2	16.3 ± 1.5	17.2 ± 1.3	15.5 ± 1.5	16.9 ± 1.1	-0.6 (-1.1 to -0.2)	0.004	negative
**BMI SDS**	0.1 ± 1.3	0.3 ± 0.9	-0.7 ± 1.1	0.1 ± 0.9	-0.7 ± 1.2	0.5 ± 0.8	-0.6 (-1.0 to -0.3)	<0.001	negative
**Total body fat (kg)**	1.0 ± 0.5	1.3 ± 0.3	1.6 ± 0.6	2.3 ± 0.7	1.8 ± 0.6	2.6 ± 1.0	-0.5 (-0.7 to -0.4)	<0.001	negative
**TBF/Weight**	0.18 ± 0.06	0.20 ± 0.04	0.18 ± 0.06	0.22 ± 0.05	0.16 ± 0.05	0.20 ± 0.06	-0.03 (-0.05 to -0.015)	<0.001	no

Data are expressed as mean ± standard deviation. TBF: total body fat.

^a^Differences and p-values were based on the comparison between VLBW children and term AGA children on average over time adjusted for gender.

^b^Negative interaction indicates that the differences between the groups became stronger over time; positive interaction indicates that the differences between the groups became less strong over time.

### IGF-I and insulin

Table 3 shows IGF-I and insulin for the VLBW and term AGA children.

**Table 3:** IGF-I and insulin.

	VLBW (n = 41)	Term AGA (n = 64)
*At 3 months (corrected) age*		
**IGF-I (nmol/l)**		7.7 (3.7–14.4)
*At 6 months (corrected) age*		
**IGF-I (nmol/l)**	10.2 (2.3–30.9)	
**Insulin (pmol/l)**	23.0 (1.0–256.7)	
*At 1 year (corrected) age*		
**IGF-I (nmol/l)**		6.8 (2.0–19.0)
**Insulin (pmol/l)**		15.7 (1.0–179.4)
*At 2 years (corrected) age*		
**IGF-I (nmol/l)**	11.6 (3.5–26.8)	9.5 (4.2–21.0)
**Insulin (pmol/l)**	21.0 (1.0–190.9)	17.9 (1.0–181.1)
*Difference between the VLBW and term AGA children on average over time adjusted for gender*		
**IGF-I**	1.36^a^ (1.19 to 1.56); p <0.001	
**Insulin**	1.37^a^ (0.86 to 2.17); p = 0.182	

Data are expressed as median and range.

^a^Difference expressed as a ratio (e.g. VLBW children have 1.36 higher concentration of IGF-I compared to term AGA children).

Longitudinal analysis showed that on average over the first 2 years of life IGF-I was significantly higher in VLBW children compared to term AGA children. Insulin did not differ between the VLBW and term AGA children over time.

In the VLBW children we also compared the early-insulin group to the standard care group and the SGA born children to the AGA born children and did not find significant differences.

In VLBW children IGF-I at 2 years corrected age was significantly higher than at 6 months corrected age (p = 0.023). In term AGA children IGF-I at 2 years of age was significantly higher than at 1 year of age (p<0.001).

### Relation of IGF-I and insulin to growth parameters

Tables 4 and 5 show the longitudinal relationship over the first 2 years of life between IGF-I and insulin on the one hand and growth parameters on the other hand. In Table 4 the relationship with the absolute growth parameters is shown and in Table 5 the relationship with the changes in growth parameters between subsequent time-points. In both groups there was a significant relationship between IGF-I and (change in) length and weight over the first 2 years of life and between IGF-I and total body fat. The relationship between IGF-I and (change in) head circumference and BMI SDS was only significant in the term AGA children.

**Table 4:** Longitudinal relationship between IGF-I and insulin and growth parameters.

	VLBW	Term AGA
**IGF-I**^**a**^		
**Weight**	0.14 (0.04 to 0.24); p = 0.004	0.23 (0.15 to 0.32); p < 0.001
**Weight SDS**	0.05 (0.01 to 0.10); p = 0.01	0.05 (0.01 to 0.08); p = 0.004
**Length**	0.58 (0.15 to 1.02); p = 0.009	0.75 (0.46 to 1.04); p < 0.001
**Length SDS**	0.06 (0.02 to 0.10); p = 0.007	-0.02 (-0.06 to 0.01); p = 0.20
**Head circumference**	0.04 (-0.08 to 0.17); p = 0.49	0.15 (0.06 to 0.24); p = 0.002
**Head circumference SDS**	0.02 (-0.01 to 0.04); p = 0.24	0.002 (-0.03 to 0.03); p = 0.90
**BMI**	0.01 (-0.05 to 0.08); p = 0.69	0.04 (-0.02 to 0.09); p = 0.20
**BMI SDS**	0.02 (-0.03 to 0.07); p = 0.41	0.08 (0.04 to 0.12); p < 0.001
**Total body fat**	0.03 (0.01 to 0.06); p = 0.006	0.06 (0.02 to 0.09); p = 0.002
**Insulin**^**b**^		
**Weight**	-0.01 (-0.02 to 0.003); p = 0.16	0.003 (-0.007 to 0.01); p = 0.56
**Weight SDS**	-0.001 (-0.01 to 0.004); p = 0.75	0.003 (-0.001 to 0.006); p = 0.09
**Length**	-0.05 (-0.11 to 0.01); p = 0.12	0.01 (-0.03 to 0.04); p = 0.65
**Length SDS**	-0.01 (-0.01 to 0.00); p = 0.05	0.006 (0.002 to 0.01); p = 0.003
**Head circumference**	-0.01 (-0.03 to 0.003); p = 0.13	-0.002 (-0.012 to 0.009); p = 0.71
**Head circumference SDS**	-0.002 (-0.01 to 0.00); p = 0.06	0.001 (-0.02 to 0.004); p = 0.55
**BMI**	0.01 (-0.002 to 0.01); p = 0.19	-0.001 (-0.01 to 0.005); p = 0.70
**BMI SDS**	0.003 (-0.003 to 0.01); p = 0.27	-0.001 (-0.006 to 0.003); p = 0.62
**Total body fat**	0.002 (-0.001 to 0.005); p = 0.26	0.008 (0.004 to 0.01); p < 0.001

All analyses were adjusted for target height, gestational age and gender.

For example, in VLBW children a difference of 1 unit in IGF-I is associated with a difference of 0.14 units in weight on average over time.

^a^Regarding IGF-I, there were significant group differences for length SDS and BMI SDS.

^b^Regarding insulin there were significant group differences for length SDS.

**Table 5:** Longitudinal relationship between IGF-I and insulin and changes in growth parameters.

	VLBW	Term AGA
**IGF-I**^**a**^		
**Δ Weight**	0.03 (0.003 to 0.06); p = 0.03	-0.01 (-0.06 to 0.04);p = 0.63
**Δ Weight SDS**	0.01 (-0.01 to 0.04); p = 0.50	0.06 (0.02 to 0.10); p < 0.001
**Δ Length**	0.15 (0.03 to 0.26); p = 0.01	0.15 (-0.30 to -0.004); p = 0.04
**Δ Length SDS**	0.01 (-0.02 to 0.04); p = 0.36	0.01 (-0.02 to 0.05); p = 0.54
**Δ Head circumference**	-0.02 (-0.05 to 0.02); p = 0.32	-0.19 (-.29 to -0.09); p < 0.001
**Δ Head circumference SDS**	0.009 (-0.01 to 0.03); p = 0.39	0.03 (-0.0036 to 0.06); p = 0.08
**Δ BMI**	-0.03 (-0.08 to 0.02); p = 0.25	-0.03 (-0.10 to 0.04); p = 0.36
**Δ BMI SDS**	-0.001 (-0.04 to 0.04); p = 0.95	0.06 (0.02 to 0.11); p = 0.01
**Δ Total body fat**	0.008 (-0.01 to 0.03); p = 0.47	-0.02 (-0.06 to 0.02); p = 0.23
**Insulin**^**b**^		
**Δ Weight**	-0.002 (-0.19 to 0.03); p = 0.16	0.005 (-0.001 to 0.01); p = 0.13
**Δ Weight SDS**	0.001 (-0.002 to 0.005); p = 0.50	0001 (-0.004 to 0.006); p = 0.64
**Δ Length**	-0.01 (-0.03 to 0.004); p = 0.14	0.02 (-0.001 to 0.03); p = 0.06
**Δ Length SDS**	0.001 (-0.003 to 0.005); p = 0.66	0.003 (-0.02 to 0.04); p = 0.54
**Δ Head circumference**	0.002 (-0.003 to 0.006); p = 0.41	0.004 (-0.008 to 0.02); p = 0.50
**Δ Head circumference SDS**	0.001 (-0.002 to 0.004); p = 0.59	0.0003 (-0.003 to 0004); p = 0.87
**Δ BMI**	0.004 (-0.003 to 0.01); p = 0.28	0.000 (-0.008 to 0.008); p = 0.99
**Δ BMI SDS**	0.001 (-0.004 to 0.007); p = 0.66	-0.001 (-0.01 to 0.004); p = 0.66
**Δ Total body fat**	0.004 (0.001 to 0.006); p = 0.02	0.007 (0.002 to 0.01); p = 0.003

All analyses were adjusted for target height, gestational age and gender.

For example, in VLBW children a difference of 1 unit in IGF-I is associated with a change of 0.03 units in weight between two subsequent measurements on average over time.

^a^Regarding IGF-I, there were significant group differences for **Δ** weight SDS, **Δ** head circumference, **Δ** BMI SDS and **Δ** total body fat.

^b^Regarding insulin there were significant group diffences for **Δ** weight and **Δ** length.

There was no significant relationship between insulin and (change in) length and weight over the first 2 years of life, but only between insulin and change in total body fat in both groups.

## Discussion

The present study shows that during the first 2 years of life VLBW infants have significantly higher IGF-I levels than term AGA children. In both VLBW and term born children there is a significant relationship between IGF-I and (change in) length and weight over the first 2 years of life.

The majority of studies concerned with IGF-I levels in VLBW infants have focused on the early postnatal period. Studies of IGF-I levels in older VLBW children are limited.

Patel et al. showed a significant correlation between IGF-I output and both weight and length, during the first two years in infants born between 24 and 33 weeks gestational age, which is in accordance with our results [20]. They did not compare IGF-I levels with a control group of term born children.

Two previous studies in mid-childhood suggest that IGF-I is involved in prolonged growth restriction in preterm born infants. Kwinta et al. showed that 7-year-old extremely-low-birth-weight children with short stature have lower IGF-I levels than those with normal stature [7]. Cutfield et al. showed that VLBW children between 5 and 10 years of age did not reach their mid-parental height SDS and had low IGF-I levels compared to term born AGA children [8].

Our present study confirms the role of IGF-I in childhood growth in VLBW infants. The VLBW children in our study were shorter than term AGA children, but the difference in length between the VLBW and term AGA children decreased during the first 2 years of life. Therefore our finding of significantly higher IGF-I levels during the first 2 years of life in VLBW children compared to term AGA children together with the longitudinal relationship between IGF-I and growth suggests that IGF-I has an important role in the catch-up growth in length in early childhood in VLBW infants. Our results are in accordance with the study of Rajaram et al., showing higher IGF-I levels in preterm infants compared to term born infants from 2 through 12 months of (corrected) age [21]. Kistner et al. found higher IGF-I levels in 9-year-old preterm born children compared to term born controls; as the preterm children were significantly shorter than the term born children, they hypothesize that preterm born children may have reduced sensitivity in the IGF-I receptors [9].

During early postnatal life and at 1 year corrected age IGF-I levels in preterm infants are positively correlated to head circumference and brain volume [22–24]. In the present study we only found a significant relationship between IGF-I and head circumference in the term AGA children. The absence of this relationship in the VLBW children is probably caused by the small size of the group. We could not explain the inverse relation between IGF-I and change in head circumference in the term AGA children.

In the study of van de Lagemaat et al. in preterm infants between term age and 6 months corrected age [25], both IGF-I and insulin were correlated to growth parameters. At 6 months corrected age the correlation between insulin and IGF-I had disappeared, indicating the shift from insulin dependency to growth hormone (GH) dependency of IGF-I with increasing postnatal age. In the present study we did not measure insulin before 6 months of (corrected) age; this explains why we could not explore any relationship between insulin and growth in our study. We hypothesize that the association between insulin and total body fat could be caused by the influence of body composition on insulin sensitivity.

We found some differences between the VLBW and term born children in the relationships between IGF-I and insulin and (changes in) growth parameters (Tables 4 and 5), but the groups are too small to draw any conclusions from these differences.

The most important limitation of our study is the small number of children. The results have to be confirmed in larger groups of VLBW children. Including serial IGF-I measurements from the early postnatal period until late childhood in both VLBW infants and term born controls at the same time points could contribute to the knowledge about the role of IGF-I in growth of VLBW infants during childhood, its relation to catch-up growth and about the meaning of the higher IGF-I levels in preterm born children compared to term born children.

Another limitation is the amount of statistical tests performed. Because of this, individual significant results should be interpreted with caution.

In conclusion, as IGF-I is related to growth in length and the difference in length compared to term born children decreases during early childhood, the higher IGF-I levels in VLBW infants during the first 2 years of life probably have an important role in catch-up growth in length.

## Supporting information

S1 Protocol Protocol local amendment to Nirture study. (PDF)
