# Supplementary material for: IGF-I and relation to growth in infancy and early childhood in very-low-birth-weight infants and term born infants
Source: PLoS One. 2017 Feb 9;12(2):e0171650. doi: 10.1371/journal.pone.0171650 (PMC5300132; doi:10.1371/journal.pone.0171650)
Supplement: S1 Protocol — (PDF) [file pone.0171650.s001.pdf]

Local amendment to the multicentre NIRTURE study

A randomised controlled trial of early insulin therapy in very low birth weight infants

(Neonatal Insulin Replacement Therapy in Europe)

Influences on growth, body composition, neuro-motor development and neuro-endocrine axes during infancy and early childhood

Department of Paediatrics

Division of Neonatology

VU University Medical Center

Protocol version 26/06/2007

Contents

Investigators........ 3

Summary of the multicentre project........ 4

Summary of the local amendment........ 6

Abbreviations........ 7

Introduction 8

Rationale........ 9

Aims of the study........ 14
- Hypothesis........ 14
- Questions........ 14

Methods
- Study population 15
- Study design 15
- Objectives........ 15
- Measurements of outcomes........ 16
- Measurements of outcomes amendment........ 16
- Assays........ 18
- Sample size consideration........ 18
- Statistical analysis........ 19

Line of investigation and collaboration........ 19

Financing........ 20

Justification........ 20

Relevance to the public........ 20

Ethical considerations........ 20

Start protocol........ 21

Consent........ 21

Future plans........ 21

References........ 23

Appendices
- Informed consent form........ 29
- Insurance........ 30
- Overview data collection........ 32
- Data laboratory assays........ 33
- Cost laboratory assays........ 34
- Patients information sheet: separate file

Investigators

Investigator	Drs. M. de Jong<br>Department of Paediatrics<br>Division of Neonatology<br>VU University Medical Center<br>Tel: 020-4442413/3013<br>e-mail: m.de.jong@vumc.nl
Supervisor	Dr. M.M. van Weissenbruch (principal investigator)<br>Department of Paediatrics<br>Division of Neonatology<br>VU University Medical Center<br>Tel: 020-4442413/3014<br>e-mail: m.vanweissenbruch@vumc.nl
Supervisor	Prof. Dr. H.N. Lafeber<br>Department of Paediatrics<br>Division of Neonatology<br>VU University Medical Center<br>Tel: 020-4442413<br>e-mail: hn.lafeber@vumc.nl
Research nurse	Mrs. A. Cranendonk<br>Department of Paediatrics<br>Division of Neonatology<br>VU University Medical Center<br>Tel: 020-4442411<br>e-mail: a.cranendonk@vumc.nl
Advisor	Dr. H.M. Dijstelbloem<br>Department of Clinical Chemistry and Endocrinology<br>VU Universitiy Medical Center<br>Tel: 020–4443872<br>e-mail: h.dijstelbloem@vumc.nl
Advisor	Prof. Dr. H.A. Delemarre-van de Waal<br>Department of Paediatrics<br>Division of Endocrinology<br>VU University Medical Center<br>Tel: 020-4440895<br>e-mail: h.delemarre@vumc.nl

Summary of the main multicentre study project

**Title**	A Randomised Controlled Trial Of Early Insulin Therapy In Very Low Birth Weight Infants
**Principal Investigator**	Professor David Dunger
**Study Sites**	Addenbrooke's Hospital, Cambridge, UK<br>New Royal Infirmary of Edinburgh, Edinburgh, UK<br>Luton and Dunstable Hospital, Luton, UK<br>Leeds General Infirmary, Leeds, UK<br>Kindergeneeskunde, U.Z., Leuven, Belgium<br>Hospital Universitari, Sant Joan de Deu, Barcelona, Spain<br>VU University Medical Center, Amsterdam, The Netherlands<br>ZOL Campus St Jan, Genk, Belgium
**Name of Product**	Insulin aspart with variable rates<br>20% dextrose support
**Dose**	0.05 units/kg/hour
**Route of administration**	Continuous intravenous infusion in the first week of life
**Study objective**	To evaluate the effect of early fixed dose insulin on mortality in very low birth weight babies
**Study Design**	Randomised controlled study.
**Study Population**	Infants with birth weight <1500g and recruited within 24 hours of birth

**Number of Patients**	500
**Multicentre**	Yes
**Number of Centres**	8 (4 UK)
**Allocation of Treatment**	Randomisation to treatment or control if all inclusion/exclusion criteria are met
**Primary Efficacy variable**	Mortality on or before expected date of delivery (EDD)
**Clinical Efficacy variable**	Improved glucose control (blood glucose 4-8 mmol/l)
**Safety Variables**	Glucose monitoring – prevalence of hypoglycaemia
**Adverse Events**	To be recorded by nursing staff on adverse event form

Summary of the local amendment

**Title**	A Randomised Controlled Trial Of Early Insulin Therapy In Very Low Birth Weight Infants; influences on growth, body composition, neuro-motor development and neuro-endocrine axes during infancy and early childhood
**Principal Investigator**	Dr. Mirjam M. van Weissenbruch
**Study Site**	VU University Medical Center, Amsterdam, The Netherlands
**Name of Product**	Insulin aspart with variable rates 20% dextrose support
**Dose**	0.05 units /kg/hour
**Route of administration**	Continuous intravenous infusion in the first week of life
**Study objective**	To evaluate the effect of early fixed dose insulin on growth, body composition, neuro-motor development, and neuro-endocrine axes during the first years of life
**Study Design**	Randomised controlled study.
**Study Population**	Infants with birth weight <1500g and recruited within 24 hours of birth
**Number of Patients**	60
**Multicentre**	No
**Allocation of Treatment**	Randomisation to treatment or control if all inclusion/exclusion criteria are met
**Primary Efficacy variable**	Improved growth, body composition, neuro-motor development and function of the neuro-endocrine axes

Abbreviations

ACTH	adrenocorticotropic hormone
AGA	appropriate for gestational age
CRH	corticotrophin releasing hormone
DEXA	dual energy x-ray absorptiometry
FSH	follicle stimulating hormone
GA	gestational age
GH	growth hormone
GH-BP	GH-binding protein
HPA	hypothalamic-pituitary-adrenal
HPG	hypothalamic-pituitary-gonadal
IGF	insulin-like growth factor
IGFBP	insulin-like growth factor-binding protein
IUGR	intrauterine growth retardation
LBW	low birth weight
LH	luteinizing hormone
SGA	small for gestational age
SDS	standard deviation score
VLBW	very low birth weight

Introduction

It is well recognized that disturbances to the fetal-maternal environment resulting in a transient restraint of prenatal growth can influence and even undermine our state of health well into adulthood. This led to the development of the fetal origins of adult disease paradigm, which resulted in a refocusing of research efforts to investigate the lifelong consequences of perinatal influences on chronic diseases and the function of neuro-endocrine axes. Meanwhile, during the last decade an abundance of data on the short- and long-term diseases associated with intrauterine growth retardation (IUGR) were published. The association between IUGR and cardiovascular disease and its risk factors (hypertension, insulin resistance) caused by the fetal adaptations to the limited supply of nutrients that lead to permanent changes in structure and metabolism (programming) is nowadays well established [1-8]. In addition, growing evidence has been documented on the relationship between IUGR and an increased risk of short stature, premature adrenarche, early puberty, polycystic ovary syndrome and associated fertility problems [9-11] suggesting that the natural history of growth, adrenal and sexual physiology may originate in the very early development, even in intrauterine life [12].

One may argue whether preterm born children may as well be predisposed to the same risks observed in children born at term after IUGR since they also go through a period of restricted growth and stress, but instead of intrauterine, like in at term born small for gestational age (SGA) children, this takes place after birth in the extra-uterine environment during a postnatal period corresponding to the last trimester. It is plausible that in this period adaptations are made that are comparable to the fetal adaptations during IUGR. These postnatal adaptations can also be the origins of disease in later life e.g. poor childhood growth, reduced insulin sensitivity in childhood, disturbances in the development and function of the hypothalamic pituitary gonadal axis (HPG-axis) and the hypothalamic-pituitary-adrenal axis (HPA-axis) and a higher risk to develop the metabolic syndrome in later life.

Our intervention, continuous insulin replacement in the first week of life (NIRTURE study) aims to improve anabolism and insulin sensitivity which may

prevent the adaptations that lead to permanent changes in organ-structure and metabolism eventually responsible for chronic diseases in adult life.

Rationale

At birth the disruption of the placental supply of nutrients leads to a period of catabolism. In term born infants usually the weight loss is maximal two or three days after birth and birth weight is recovered after seven days. In very low birth weight infants (<1500g) this period of catabolism is much more prolonged and birth weight is often not regained for several weeks. VLBW infants have a high incidence of hyperglycaemia, caused by a combination of insulin resistance and relative insulin deficiency.

There is increasing evidence that not only the prenatal period but also the early postnatal period is critical for pancreatic development. At this time a complex set of signals appears to influence pancreatic development and ß cell survival. This has implications both in terms of acute glucose control but also relative insulin deficiency is likely to play a role in poor postnatal growth, which has been associated with later motor and cognitive impairment, and fewer ß cells are linked to risk of type 2 diabetes later in life.

Recent studies show that changes in metabolic and endocrine processes because of a period of catabolism pre- or postnatally are not only profound in the early postnatal period but also linked to chronic diseases in later life. Infants born either prematurely or SGA because of IUGR are at increased risk of poor childhood growth and reduced insulin sensitivity in childhood also leading to a higher risk of the metabolic syndrome in later life [13-17]. With respect to this, it is important to mention that the underlying mechanisms leading to poor childhood growth and reduced insulin sensitivity are far from clear. There are indications that especially SGA born prepubertal children who did catch up in height and/or weight during the first years of life, i.e. not showing a poor childhood growth, have a reduced insulin sensitivity [13].
One can imagine, that during the postnatal period and even during childhood there are not only disturbances in growth but there may also be disturbances in body composition. Cooke et al. [19] investigating body composition during the

first year of life in preterms and infants born at term did not found differences in body composition between the groups despite the preterms studied were smaller. Also Bolt et al. [18] in an observational cohort study of preterms did, however, not observe differences in body composition relative to body weight between AGA and SGA infants during the first year of life, despite differences in catch up growth.

Our intervention aims to improve anabolism and insulin sensitivity during the first week of life. As early growth is increasingly being shown to be important in terms of long-term metabolism [20-23], it is important that we review these children in early childhood to assess their growth, body composition and insulin sensitivity.

In extremely preterm infants and SGA infants born at term Growth Hormone (GH) resistance is found. These infants have higher levels of GH and insulin-like growth factor binding protein-1 (IGFBP-1) and low levels of insulin-like growth factor-1 (IGF-1) and IGFBP-3. Both IGF-1 and IGFBP-3 are positively related to growth. Low levels might be one of the causes of the slow postnatal growth in VLBW infants.

Levels of IGF-1 and the inhibitory IGF binding protein IGFBP-1 and IGFBP-3 are regulated by insulin in the newborn [24-26]. Insulin stimulates IGF-1 and IGFBP-3 synthesis and inhibits IGFBP-1 synthesis [27]. It is well established that preterm infants are at high risk of developmental delay and early growth has also been linked to neuro-developmental outcome. IGF-I has an important role in fetal and postnatal brain growth [27-28]. Furthermore, low IGF-1 levels have been implicated in the pathogenesis of retinopathy of prematurity [29-31]. Thus theoretically, improved insulin delivery and restoration of IGF-I levels could have important implications for the long-term outcome as well as the short-term growth of very low birth weight babies.

In early life there are differences in the development and function of the HPA-axis between preterm and term born infants and between AGA and SGA preterm infants. During gestation the HPA-axis develops and in the third trimester of gestation this axis fully matures. The adrenal cortex function in preterm infants is

closely related to the duration of gestation. Bolt et al. showed that AGA preterms born < 30 weeks of gestation had a lower response of cortisol to ACTH stimulation on the 5th to 10th day of life compared to AGA preterms born between 30 and 33 weeks [32]. In preterm infants birth weight SD score as a measure for intrauterine growth was positively associated with the cortisol response to ACTH stimulation [33]. The lower adrenal response to stimulation may be involved in the higher risk of neonatal morbidity in very preterm infants, and especially in growth restricted preterm infants. In agreement with this, Scott et al. found that plasma cortisol levels in preterm infants not only significantly correlated with gestational age but also with markers of illness [34].
In conclusion, prematurity and fetal growth restriction have consequences for the function of the HPA-axis in the neonatal period and this is probably associated with neonatal morbidity.

With respect to morbidity in adult life one of the proposed mechanisms underlying the associations between intrauterine growth restriction and adult cardiovascular disease and its risk factors (hypertension, insulin resistance) is intrauterine programming of the HPA-axis. Phillips et al. found an inverse relation between birth weight and fasting plasma cortisol in adults. These cortisol concentrations correlated positively to systolic blood pressure, fasting and 2-hour plasma glucose concentrations after an oral glucose tolerance test, plasma triglyceride levels and insulin resistance [35,36]. Even in prepubertal SGA children born at term compared to AGA children born at term salivary cortisol concentrations during the day were higher, and therefore indicative of increased HPA-activity [37].

Case control studies comparing small for gestational age to normal birth weight children born at term show that in contrast to the low DHEAS levels at birth, DHEAS levels are higher than average in older low-birth weight children [10,13,38,39]. Recently, a large birth cohort study of unselected UK subjects (ALSPAC) showed a rather continuous inverse relationship between birth weight and DHEAS levels throughout the range of birth weights [40], comparable to the relationships between birth weight and disease risk in adulthood [41].

The increase in DHEAS levels seems to follow the typical pattern of rapid early postnatal weight gain that is seen in the majority of low birth weight children. There are also studies indicating that increased adrenal adrogen secretion during childhood may be programmed by the combination of reduced fetal growth and rapid early postnatal weight gain [42]. In particular the rapid weight gain during the first 3 years of life appears to influence the onset of adrenarche [43], suggesting that DHEAS could be regulated by weight gain. Precocious or premature adrenarche refers to an early increase in adrenal androgen production secondary to an early isolated maturation of the adrenal gland [44-49]. Premature adrenarche generally occurs with increasing frequency between the ages of 3 and 8 yr, although it may present as early as 6 months of age [50]. IGF-1 and insulin levels are higher in children with premature adrenarche than in control children [50-52], and might therefore link the combination of low birth weight and rapid infancy weight gain to the development of higher adrenal androgen production in later life [9,10,13,53].
Clinical features of adrenal hyperandrogenism are associated with adverse effects on body composition, insulin resistance, and increased risk of future progression to ovarian hyperandrogenism in the early years post-menarche.

Premature born children also go through a period of restricted growth and stress; it is plausible that the adaptations made in this early neonatal period can also be the origins of adrenal hyperandrogenism and future diseases in later life.

Puberty, defined as the achievement of interactions between the neuro-endocrine unit, the gonads and the hypothalamic-pituitary-gonadal (HPG)-axis, has its origin in the fetal period, when this endocrine system is already active. This endocrine unit becomes suppressed during infancy (3-6 months after birth) and is reactivated at the onset of puberty.
Over the last decade, growing evidence has also been documented on the relationship between IUGR in term born children and pubertal development, indicating changes in timing and progression of puberty [9,54,55]. Both the role of IUGR and the mechanisms behind the onset and progression of puberty are still elusive. Based on the knowledge that the HPG-apparatus in the human fetus is functional by 50 days of gestation, one can postulate that the maturational

process of this endocrine system that progresses even until 3 to 6 months after birth can be influenced in periods of restricted growth both in-utero and ex-utero. As premature born children also go through a period of restricted growth and stress, it is plausible that prematurity has also consequences for the development of the HPG-axis and later puberty.

Insulin replacement therapy

Our intervention, continuous insulin replacement in the first week of life (NIRTURE study) aims to improve anabolism and insulin sensitivity, which may prevent the adaptations that lead to permanent changes in structure and metabolism.

As early growth is increasingly being shown to be important in terms of long-term metabolism [20-23] it is important to investigate their growth, body composition, insulin sensitivity and neuro-motor and neuro-endocrine development at least postnatally and in early childhood.

Aims of the study

In the main multi-centre study 500 preterm infants will be randomised to receive either a continuous infusion of insulin for the first 7 days of life, or receive standard neonatal care. Aims of this amendment study are to investigate in a subpopulation of 60 preterms the effect of this early insulin replacement on growth and body composition, insulin sensitivity, the function of the HPA-axis and HPG-axis and neuro-motor development in infancy and during early childhood.

Hypothesis

Relative insulin deficiency in the very low birth weight baby leads to profound catabolism, insulin resistance and hyperglycaemia during the first week of life. We hypothesize that insulin replacement may improve weight gain and IGF-I generation that could have implications for (brain) growth, body composition and later insulin sensitivity, neuro-endocrine and neuro-motor developmental outcomes by preventing the adaptations leading to permanent changes in organ structure and metabolism.

Questions:

Primary questions:

1. Does insulin replacement in preterm infants during the first week of life improve insulin sensitivity, early postnatal growth, catch up growth and body composition during the first years of life?
2. Does insulin replacement during the first week of life have implications for the development and function of the HPA and HPG axis?
3. Does insulin replacement during the first week of life have implications for the neuro-motor development during infancy and early childhood?

Secondary questions:

1. Is insulin sensitivity related to early postnatal growth, catch up growth and body composition in early childhood?

Methods

Study population

Preterm infants that are included in the NIRTURE study will be included in this study indicating similar inclusion and exclusion criteria as in the NIRTURE study.

Inclusion criteria:

- birth weight < 1500g
- less than 24 hours of age
- requiring intensive care and in whom it is considered appropriate to continue intensive care
- written informed parental consent

Exclusion criteria

- maternal diabetes including gestational diabetes
- babies where the appropriateness of continuing intensive care is being discussed
- major congenital anomalies

Design

Randomised controlled study

Objectives

Primary:

1. Growth and catch up growth and body composition during the first two years corrected age

Secondary

1. Insulin sensitivity at the age of 6 months and 2 years
2. Adrenal function at the age of 6 months and 2 years
3. Development of the HPG axis postnatally until the age of 3 months
4. Neuro-motor and developmental outcome during the first 2 years corrected age

Measurements of outcomes

Study design NIRTURE study (briefly)

In the multi-centre randomised controlled trial, VLBW infants (birth weight <1500g) will be randomised within 24 hours of delivery to either treatment with early fixed dose insulin (0,05 E/kg/hr) with 20% dextrose to maintain normoglycaemia, or to receive standard neonatal care.

Those randomised to treatment will receive a fixed dose of insulin combined with variable 20% dextrose support throughout the first week. Additional insulin will be infused if blood glucoses are consistently above 12 mmol/l, and an infusion of 20% dextrose will be started or increased if blood glucose falls to <4 mmol/l to prevent hypoglycaemia (blood glucose <2,6 mmol/l). Controls will receive standard neonatal care.

All babies will be monitored using a Minimed continuous glucose monitor for 7 days. Blood samples for measurement of IGF-1 (bioassay), IGFBP-1 and cytokines are taken on day 1, 3, 7 and 28 and a urine sample for measurement of 3-methylhistidine/creatinine ratio will be collected on day 7; all these samples are sent to Cambridge for analysis. Clinical data are recorded and after discharge all patients are followed in the outpatient clinic.

Measurement of outcomes amendment

Growth and body composition

- Anthropometry according to Dauncey et al. [57] on day 7, at 32 weeks postmenstrual age, at expected date of delivery (40 weeks) and at the corrected age of three, six, twelve and twenty-four months. Measurements include naked body weight (measured on a electronic scale to the nearest 5 g), crown-heel length (measured on a length board to nearest 1 mm) and head circumference (using a measuring tape to the nearest 1 mm). Crown-back length and bilateral lengths of the upper arms, lower arms and lower legs are measured (total length). In addition, chest circumference, bilateral circumferences of the upper arms, thighs and calves (total circumference) and bilateral skin-fold thickness at four sites (subscapular, abdominal, triceps and biceps) are measured.

- quantitative ultrasound of the second metacarpus [59] for assessment of bone mineral status on day 7, at 32 weeks postmenstrual age, at expected date of delivery (40 weeks) and at the corrected age of three, six, twelve and twenty-four months
- IGFBP-3 on day 7 and 28 and IGF-1, IGFBP-1 and IGFBP-3 in serum at the corrected age of 6 and 24 months

2. Insulin sensitivity
   - fasting glucose and insulin in plasma and calculation of insulin sensitivity using the HOMA method [60] at the corrected age of 6 and 24 months
3. Adrenal function
   - cortisol measurement in saliva at 24 months corrected age
   - DHEAS will be measured in plasma and saliva at the corrected age of 6 and 24 months corrected age.
4. Blood pressure
   - blood pressures: these are collected as part of the clinical data during hospital stay and will also be measured during follow-up in the outpatient clinic at 24 months corrected age
5. Gonadotrophins and steroids
   - gonadotrophin (FSH and LH) measurements in urine on day 7 and 28, at 32 weeks postmenstrual age, at expected date of delivery (40 weeks) and at the corrected age of three months. As shown by Kuijper et al. urinary gonadotrophin levels are a proper reflection of the serum levels [61].
   - oestrogens and androgens in urine on day 7 and 28, at 32 weeks postmenstrual age, at expected date of delivery (40 weeks) and at the corrected age of three months
6. Neurodevelopment during the first two 2 years of life according to routine follow up of preterms in the VUmc
   - developmental tests: standardised neurological assessment according to Prechtl at expected date of delivery (0 months) and according to Touwen at the corrected age of 3, 6, 12 and 24 months [62,63,64]. Alberta Infant Motor Scale (AIMS) [65] and the motor Bayley Scales at a corrected age of 6 and 12 months. Psychomotor development indices

and mental development indices at 12 and 24 months corrected age. Current method used is the Bayley Scales of Infant Development II (BSID-II) Motor Scale [66]

Assays

Blood sample analysis:

Blood samples collected will be stored at -80ºC until IGF-1/IGFBP-1/IGFBP-3/ insulin/ DHEAS are determined by immunoassay (see page 32).
Glucose will be measured by the hexokinase method.

Saliva sample analysis:

Saliva samples collected will be stored at -20ºC until DHEAS and Cortisol are determined by immunoassay (see page 32).

Urine sample analysis

Urine samples are collected in a pediatric urine collection pouch and will be stored at -20ºC until FSH and LH are determined.
Gonadotrophin levels will be measured by the Architect method using reagents obtained from Abbott Laboratories (Diagnostics Division Abbott Park, Illinois, USA) (see page 32).

Oestrogens (ERα CALUX) and androgens (AR CALUX) levels in urine will be measured using the Chemically Activated LUciferase eXpression (CALUX) reporter gene assays [67,68].

Sample size consideration

Bolt et al [56] studied growth and body composition in 15 SGA and 26 AGA preterm born infants during the first 2 years of life. They found that growth expressed as anthropometry according to Dauncey [57], IGF-1 and IGFBP-3 were significantly lower in SGA than in AGA infants ($p<0.01$). Catch up growth during the first year of life was significantly higher in SGA infants compared to AGA infants ($p<0.01$).
In addition, growth and body composition was studied in 14 preterm infants with chronic lung disease and treated with corticosteroids to facilitate weaning from

the ventilator and 18 preterm controls during the first 2 years of life [58]. Body composition at term and 3 months corrected age in preterm infants treated with dexamethasone for chronic lung disease, who received extra caloric intake until term age, did not differ from preterm infants without CLD.
Based on the results of both studies in combination with power calculation it is expected that groups of 30 fully evaluable subjects (insulin treated vs. controls) will be sufficient to detect a difference of 0.5 SDS in growth parameters with a power of 80% at $\alpha=0.05$.

Statistical analysis

Results are presented as mean ± standard deviation. We will compare the results of infants treated with insulin to those receiving standard care on an intention to treat basis and within each group we will also compare the results of SGA infants to AGA infants.
Comparison between the groups on all different time points will be analysed using the unpaired t-test.
Within group differences between different time points and differences between the groups in the changes of the values in time will be analysed with repeated measures analysis of variance (ANOVA).
A p value of <0.05 will be considered as statistically significant

Line of investigation and collaboration

The current research is part of the research theme “neuro-endocrine regulation systems, growth and metabolism” of the Institute of Clinical and Experimental Neurosciences (ICEN-VUmc). Several studies have already been started in this specific line of investigation. The subjects for the local study are from the multicentre NIRTURE study.

Financing

Since Novo Nordisk Nederland is interested in the long term effects of early treatment with insulin, we will apply for an extra funding for this extension.

Justification

Infants born either prematurely or small for gestational age are at increased risk of poor childhood growth, reduced insulin sensitivity in childhood and higher risk of the metabolic syndrome in later life. Our intervention aims to improve anabolism, insulin resistance and hyperglycaemia during the first week of life. Insulin deficiency may contribute to slow weight gain and impaired IGF-I generation which could have implications for risk of retinopathy, brain growth and later neurodevelopmental outcomes. It is important therefore that we assess the longer-term impact of our intervention in terms of growth, body composition and insulin resistance in later childhood, as well as neurodevelopmental outcome.

Relevance to the public

Many children are born preterm: one percent of children is born before 32 weeks gestational age. The burden of caring for preterm infants is potentially large both in terms of the costs of the initial neonatal intensive care, but also longer term societal costs. It is therefore important to ascertain whether the additional short term costs of intensive insulin therapy are justified by any improvement in outcomes (e.g. morbidity at one year) or reduction in the longer term costs of hospitalisation or community health services.

Ethical considerations

Taken the blood samples of the first project into account only at the corrected age of 6 and 24 months extra blood samples (total amount 1 ml) will be taken. These samples have to be taken prior to feeding. The sample size at the age of 7 days and 28 days for the first project will be increased with 250 μl; these blood samples are taken from the arterial line. Insertion of a central venous and arterial line in these very low birth weight infants is part of routine care.
Taken the urine sample of the first project into account, 4 extra urine samples (5 ml) will be taken on day 28, at 32 weeks postmenstrual age and at the corrected age of 0 and 3 months. The sample size at the postnatal age of 7 days will be

increased with 5 ml. All urine samples are collected in a pediatric urine collection pouch.
It is important to notice that in this project most determinations in blood and urine are accomplished in samples that are already collected for clinical reasons; the remaining material is stored and used for analysis within this project.
Anthropometry and quantative ultrasound will be performed on day 7, at 32 weeks postmenstrual age and at the corrected age of 0, 3, 6, 12 and 24 months. During hospitalisation this will be combined with routine nursing care to minimize the burden.
Saliva samples will be collected at the corrected age of 6 and 24 months.
Data of each subject will be stored in a case record form (CRF) and into a computer file for analysis. Patient data, blood, urine and saliva samples will be anonymised. After analysis, the data will be used for scientific presentations and publications in scientific journals. All data will be reported anonymously.
Unexpected findings will be reported to the pediatrician and the general practitioner in writing. The subjects will be insured according to the research insurance policy of the VU University medical center (see appendices).

Start protocol

This protocol will be started as soon as all required permissions have been obtained.

Consent

Parents will be given an information sheet prior to attendance for the whole project. Written informed parental consent will then be obtained prior to participation.

Future plans

Although brain neurons growth and differentiate for the major part during the fetal period, neuronal growth and plasticity still occur during childhood. The process of maturation and myelinisation of long distance connections between the different brain areas is said to continue even up to the age of 30 years.

It has been described that infants born with a low birth weight for gestational age due to last trimester growth failure are at increased risk for later mild cognitive deficits and behavioural problems, particularly difficulties in attention and hyperactivity, compared to controls born appropriate for gestational age (69,70,71).
Also young adults born very prematurely and/or with a very low birth weight had moderate and severe problems in cognitive and neurosensory functioning. Compared to the general Dutch population twice as many young adults born very prematurely and/or with a very low birth weight were poorly educated and three times as many were neither employed nor in school at age 19 (72).
Whether cognitive functions in preterms are impaired due to inappropriate developmental outgrowth after birth is not known. This is also the case for the fact whether early insulin treatment is capable to stimulate neuronal outgrowth and to increase IQ's.
It is expected that neuropsychological assessment and functional imaging techniques above the age of 5 years might increase our understanding on the impact of early insulin therapy on higher brain functioning in children born preterm. It is planned to investigate this in a next amendment on this cohort of preterms.

References

1. Hales CN, Barker DJ, Clark PM, Cox LJ, Fall C, Osmond C et al. Fetal and infant growth and impaired glucose tolerance at age 64. BMJ 1991; 303(6809):1019-1022.

2. Barker DJ. Fetal origins of coronary heart disease BMJ 1995;15:311(6998):171-4. Review

3. Barker DJ. In utero programming of chronic disease. Clin Sci 1998;95:115-28.

4. Barker DJ. Fetal origins of cardiovascular disease. Ann Med 1999;31 suppl 1:3-6.

5. Curhan GC, Willett WC, Rimm EB, Spiegelman D, Ascherio AL, Stampfer MJ. Birth weight and adult hypertension, diabetes mellitus, and obesity in US men. Circulation 1996; 94(12):3246-3250.

6. Lithell HO, McKeigue PM, Berglund L, Mohsen R, Lithell UB, Leon DA. Relation of size at birth to non-insulin dependent diabetes and insulin concentrations in men aged 50-60 years. BMJ 1996; 312(7028):406-410.

7. McCance DR, Pettitt DJ, Hanson RL, Jacobsson LT, Knowler WC, Bennett PH. Birth weight and non-insulin dependent diabetes: thrifty genotype, thrifty phenotype, or surviving small baby genotype? BMJ 1994; 308(6934):942-945.

8. Phipps K, Barker DJ, Hales CN, Fall CH, Osmond C, Clark PM. Fetal growth and impaired glucose tolerance in men and women. Diabetologia 1993; 36(3):225-228.

9. Van Weissenbruch MM, Delemarre-van de Waal HA. Early influences on the tempo of puberty. Horm Res 2006;65(suppl 3):105-111.

10. Veening MA, van Weissenbruch MM, Roord JJ, Delemarre-van de Waal HA. Pubertal development in children born small for gestational age.J Pediatr Endocrinol Metab 2004; 17:1497-1505.

11. Ibanez L, De Zegher F. Puberty and prenatal growth. Molecular and Cellular Endocrinology 2006; 254-255:22-25.

12. Fowden AL, Forhead AJ. Endocrine mechanisms of intrauterine programming. Reproduction 2004; 127: 515-526.

13. Veening MA, Van Weissenbruch MM, Delemarre-Van De Waal HA. Glucose tolerance, insulin sensitivity, and insulin secretion in children born small for gestational age. J Clin Endocrinol Metab 2002;87:4657-61.

14. Hofman PL, Regan F, Jackson WE, Jefferies C, Knight DB, Robinson EM, Cutfield WS. Premature birth and later insulin resistance. N Engl J Med 2004;351:2179-86.

15. Bazaes RA, Alegria A, Pittaluga E, Avila A, Iniguez G, Mericq V. Determinants of insulin sensitivity and secretion in very-low-birth-weight children. J Clin Endocrinol Metab 2004;89:1267-72.

16. Finken MJ, Keijzer-Veen MG, Dekker FW, Frolich M, Hille ET, Romijn JA, Wit JM; on behalf of the Dutch POPS-19 Collaborative Study Group. Preterm birth and later insulin resistance: effects of birth weight and postnatal growth in a population based longitudinal study from birth into adult life Insulin resistance 19 years after preterm birth. Diabetologia 2006;49:478-85.

17. Irving RJ, Belton NR, Elton RA, Walker BR. Adult cardiovascular risk factors in premature babies. Lancet 2000; 355(9221):2135-2136.

18. Bolt RJ, Weissenbruch MM, Lafeber HN, Delemarre-van de Waal. Growth and body composition in small for gestational age preterm infants during infancy. submitted

19. Cooke RJ, Rawlings DJ, McCormick K, Griffin IJ, Faulkner K, Wells JC, Smith JS, Robinson SJ. Body composition of preterm infants during infancy. Arch Dis Child Fetal Neonatal Ed 1999;80:F188-91.

20. De Curtis M, Rigo J. Extrauterine growth restriction in very-low-birthweight infants. Acta Paediatr 2004;93(12):1563-8.

21. Singhal A, Cole TJ, Fewtrell M, Deanfield J, Lucas A. Is slower early growth beneficial for long-term cardiovascular health? Circulation 2004; 109(9):1108-13.

22. Singhal A, Fewtrell M, Cole TJ, Lucas A. Low nutrient intake and early growth for later insulin resistance in adolescents born preterm. Lancet 2003;361(9363):1089-97.

23. Fitzhardinge P, Inwood S. Long-term growth in small for date children. Acta Paediatr Scand (suppl) 1989;349:27-33.

24. De La Puente A, Goya L, Ramos S, et al. Effects of experimental diabetes on renal IGF/IGFBP system during neonatal period in the rat. Am J Physiol Renal Physiol 2000;279(6):F1067-76.

25. Ogilvy-Stuart AL, Hands SJ, Adcock CJ, et al. Insulin,insulin-like growth factor 1(IGF-1), IGF-binding protein-1, growth hormone, and feeding in the newborn. J Clin Endocrinol Metab 1998;83(10):3550-3557.

26. Goya L, de la Puente A, Ramos S, et al. Regulation of IGF-I and -II by insulin in primary cultures of fetal rat hepatocytes. Endocrinology 2001;142(12):5089-96.

27. Dentremont KD, Ye P, D'Ercole AJ, O'Kusky JR. Increased insulin-like growth factor-I (IGF-I) expression during early postnatal development differentially increases neuron number and growth in medullary nuclei of the mouse. Brain Res Dev Brain Res 1999;114(1):135-41.

28. Davidson S, Shtaif B, Gil-Ad I, et al. Insulin, insulin-like growth factors-I and -II and insulin-like growth factor binding protein-3 in newborn serum: association with normal fetal head growth and head circumference. J Pediatr Endocrinol Metab 2001;14(2):151-8.

29. Hikino S, Ihara K, Yamamoto J, et al. Physical growth and retinopathy in preterm infants: involvement of IGF-I and GH. Pediatr Res 2001;50(6):732-6.

30. Hellstrom A, Carlsson B, Niklasson A, et al. IGF-I is critical for normal vascularization of the human retina. J Clin Endocrinol Metab 2002; 87(7):3413-6.

31. Hellstrom A, Perruzzi C, Ju M, et al. Low IGF-I suppresses VEGF-survival signaling in retinal endothelial cells: direct correlation with clinical retinopathy of prematurity. Proc Natl Acad Sci U S A 2001;98(10):5804-8.

32. Bolt RJ, Van Weissenbruch MM, Popp-Snijders C, Sweep FG, Lafeber HN, Delemarre-van de Waal HA. Maturity of the adrenal cortex in very preterm infants is related to gestational age. Pediatr Res 2002;52:405-10.

33. Bolt RJ, van Weissenbruch MM, Popp-Snijders C, Sweep CG, Lafeber HN, Delemarre-van de Waal HA. Fetal growth and the function of the adrenal cortex in preterm infants. J Clin Endocrinol Metab 2002;87:1194-9.

34. Scott SM, Watterberg KL. Effect of gestational age, postnatal age, and illness on plasma cortisol concentrations in premature infants. Pediatr Res 1995;37:112-6.

35. Phillips DI, Barker DJ, Fall CH, Seckl JR, Whorwood CB, Wood PJ, Walker BR. Elevated plasma cortisol concentrations: a link between low birth weight and the insulin resistance syndrome?. J Clin Endocrinol Metab 1998;83:757-60.

36. Phillips DI, Walker BR, Reynolds RM, Flanagan DE, Wood PJ, Osmond C, Barker DJ, Whorwood CB. Low birth weight predicts elevated plasma cortisol concentrations in adults from 3 populations. Hypertension 2000;35:1301-6.

37. Veening MA, van Weissenbruch MM, Delemarre-van de Waal HA. Ambulatory blood pressure and salivary cortisol concentrations in children born small for gestational age. submitted.

38.Franks S. Adult polycystic ovary syndrome begins in childhood. Best Pract Res Clin Clin Endocrinol Metab 2002;16:263-272.

39.Franks F. Polycystic ovary syndrome. N Engl J Med 1995;333:853-861.

40.Ong KK, Potau N, Petry CJ, Jones R, Ness AR, Honour JW, de Zegher F, Ibanez L, Dunger DB. Opposing influences of prenatal and postnatal weight gain on adrenarche in normal boys girls. J CLin Endocrinol Metab 2004;89:2647-2651.

41.Hales CN, Barker DJ. The thrifty phenotype hypothesis. Br Med Bull 2001;60:5-20.

42.Ong K. Adrenal function in low-birth-weight children. Endocr Dev. Basel Karger 2005, Vol 8:pp 34-53.

43.Ong KK, Ahmed ML, Emmett PM, Preece MA, Dunger DB, the ALSPAC study team. Association between postnal catch-up growth and obesity in childhood: prospective cohort study. BMJ 2000;320:967-971.

44.Korth-Schutz S, Levine LS, New MI. Serum androgens in normal prepubertal and pubertal children with precocious adrenarche. J Clin Endocrinol Metab 1976;42:117-124.

45.Reiter E, Fuldauer VG, Root AW. Secretion of the adrenal androgen, dehydroepiandrosterone sulfate during normal infancy, childhood and adolescence, in sick infants and in children with endocrinologic abnormalities. J Pediatr 1977;90:766-770.

46.Rosenfield RL, Rich BH, Lucky AW. Adrenarche as a cause of benign pseudopuberty in boys. J Pediatr 1982;101:1005-1009.

47.Parker LN, Sack J, Fisher DA, Odell WD. The adrenarche:prolactin, gonadotropins, adrenal androgens and cortisol. J Clin Endocrinol Metab. 1978;46:396-401.

48.Voutilainen R, Perheentupa j, Apter D. Benign premature adrenarche: clinical features and serum steroid levels. Acta Paediatr Scand 1983; 72:707-711.

49.Silverman SH, Migeon CJ, Rosenberg E, Wilkins L. Precocious growth of sexual hair without other secundary sexual development:”premature pubarche”a constitutional variation of adolescence. Pediatrics 1952;10:426-432.

50.Ibanez L, Potau N, Zampolli M, Rigue S, Saenger P, Carrascosa A. Hyperinsulinemia and decreased insulin-like growth factor-binding protein-1 are common features in prepubertal and pubertal girls with a history of premature pubarche. J Clin Endocrinol Metab 1997;82:2283-2288.

51. Silfen ME, Manibo AM, Ferin M, McMahon DJ, Levine LS, Oberfield SE. Elevated free IGF-1 levels in prepubertal Hispanic girls with premature adrenarche: Relationship with hyperandrogenism and insulin sensitivity. J Clin Endocrinol Metab 2002;87:398-403.

52. Denburg MR, Silfen ME, Manibo AM, Chin D, Levine LS, Ferin M, McMahon DJ, Go C, Oberfield SE. Insulin sensitivity and the insulin-like growth factor system in prepubertal boys with premature adrenarche. J Clin Endocrinol Metab 2002;87:5604-5609.

53. Zhang LH, Rodriquez H, Ohno S, Miller WL. Serine phosphorylation of human P450c17 increases 17,20-lyase activity: Implications for adrenarche and the polycystic ovary syndrome. Proc Natl Acad Sci USA 1995;92:10619-10623.

54. De Zegher F, Ibanez L. Novel insights into the endocrine-metabolic and reproductive consequences of prenatal growth restraint in girls. Girls-born-small become women-born-small. Verh K Acad Geneeskd Belg 2004;66:353-82.

55. Ibanez L, Ferrer A, Marcos MV, Hierro FR, de Zegher F. Early puberty: rapid progression and reduced final height in girls with low birth weight. Pediatrics 2000;106:72.

56. Bolt RJ, van Weissenbruch MM, Lafeber HN, Delemarre-van de Waal HA. Growth and body composition in small for gestational age preterm infants during infancy.J Pediatric Gastroenterology & Nutrition 2004;39 (1):S416-S417.

57. Dauncey MJ, Gandy G, Gairdner D. Assessment of total body fat in infancy from skin-fold thickness measurements. Arch Dis Child 1977;52:223-7.

58. Bolt RJ, Van Weissenbruch MM; Roos JC, Delemarre-Van De Waal HA, Cranendonk A, Lafeber HN.Body composition in infants with chronic lung disease after treatment with dexamethasone. Acta Paediatrica 2002; 1(7):815-821.

59. Ritschl E, Wehmeijer K, DE Terlizzi F, Wipfler E, Cadossi R, Douma D, Urlesberger B, Muller W.Assessment of skeletal development in preterm and term infants by quantitative ultrasound. Pediatr Res 2005;58:341-6.

60. Matthews DR, Hosker JP, Rudenski AS, Naylor BA, Treacher DF, Turner RC. Homeostasis model assessment: insulin resistance and beta-cell function from fasting plasma glucose and insulin concentrations in man. Diabetologia 1985; 28(7):412-419.

61. Kuijper EA, Houwink EJ, van Weissenbruch MM, Heij HA, Blankenstein MA, Huijser J, Martens F, Lambalk CB. Urinary gonadotropin measurements in neonates: a valuable non-invasive method. Ann Clin Biochem 2006; 43:320-2.

62.Prechtl HFR, Beintema D. The neurological Examination of the full-term newborn infant. London: Spastics International Medical Publications/ William Heinemann Medical Books, 1964.

63.Touwen BCL. Neurological development in infancy. London: Clinics in Developmental Medicine, 1976.

64.Touwen BC, Huisjes HJ, Jurgens-van der Zee AD, Bierman-van Eendenburg ME, Smrkovsky M, Olinga AA. Obstetrical condition and neonatal neurological morbidity. An analysis with the help of the optimality concept. Early Hum Dev 1980;4(3):207-228.

65.Piper MC, Darrah J. Motor assessment of the developing infant. Philadelphia, Pa:WB Saunders Co.;1994.

66.van der Meulen BF, Ruiter SAJ, Lutje Spelberg HC, Smrkovsky M. BSID-II-NL Bayley scales of infant development-second edition-nederlandse versie.

67.Sonneveld E, Riteco JAC, Jansen HJ, Pieterse B, Brouwer A, Schoonen WG, van der Burg B. Comparison of In Vitro and In Vivo screening models for androgenic and estrogenic activities. Toxicological Sciences 2006 ;89 (1): 173187.

68.Sonneveld E, Jansen HJ, Riteco JAC,Brouwer A, van der Burg B. Developmentof androgen- and estrogen responsive bioassays, members of a panel of human cell line-based highly selective steroid-responsive bioassays. Toxicological sciences 2005;83:136-148.

69.Viggedal G, Lundalv E, Carlsson G, Kjellmer I. Neuropsychological follow-up into young adulthood of term infants born small for gestational age. Med Sci Monit 2004;10(1):CR8-16.

70.Goldenberg RL, Hoffman HJ, Cliver SP. Neurodevelopmental outcome of small-for-gestational-age infants. Eur J Clin Nutr 1998;52 suppl 1:S54-S58.

71.Pryor J, Silva PA, Brooke M. Growth, development and behaviour in adolescents born small for gestational age. .J Paediatr Child Health 1995;31(5):403-407.

72.Elysee TM Hille et all for the Dutch Collaborative POPS 19 Study Group. Functional outcomes and participation in young adulthood for very premature and very low birth weight infants: The Dutch POPS-study at 19 years of age. Accepted for publication in Pediatrics sept 2007 issue.

A Randomised Controlled Trial of Early Insulin Therapy in Very Low Birth Weight Infants

(vroege insulinebehandeling bij premature pasgeborenen, een gerandomiseerd gecontroleerd onderzoek)
met lokaal amendement: onderzoek naar de groei en lichaamssamenstelling, de psychomotore en neuro-endocriene ontwikkeling

<u>Toestemmingsformulier</u>

('informed consent')
Ik verklaar hierbij op voor mij duidelijke wijze, mondeling en schriftelijk te zijn ingelicht over aard, methode en doel van bovengenoemd onderzoek.
Mijn vragen zijn naar tevredenheid beantwoord. De schriftelijke informatie, behorend bij deze verklaring, is mij overhandigd. Ik stem geheel vrijwillig in met deelname van mijn zoon/dochter aan dit onderzoek. Ik behoud daarbij het recht deze instemming weer in te trekken zonder dat ik daarvoor een reden hoef op te geven.

Naam patiënt: …………………………………………..

Naam moeder: ………………………………………….   ………………………….…. (handtekening)

Naam vader: ……………………………………………..   ……………………………... (handtekening)

Amsterdam, ………………………………………………… (datum)

Ik heb mondeling en schriftelijk toelichting verstrekt op het onderzoek. Ik verklaar mij bereid nog opkomende vragen over het onderzoek te beantwoorden. Indien u tijdens het onderzoek besluit dat uw zoon/dochter niet verder meedoet aan het onderzoek zal deze beslissing geen invloed hebben op de (na)zorg waar iedere patiënt in dit ziekenhuis recht op heeft.

Naam arts: …………………………….………………

Handtekening: ……………………………………….……

Amsterdam,   ……..…………………………………… (datum)

TOELICHTING VERZEKERING

Titel onderzoek: A Randomised Controlled Trial Of Early Insulin Therapy In Very Low Birth Weight Infants (vroege insulinebehandeling bij premature pasgeborenen, een gerandomiseerd gecontroleerd onderzoek)

De opdrachtgever voor bovengenoemd wetenschappelijk onderzoek, het VU medisch centrum,
heeft u verzekerd in verband met eventuele schade die u zou kunnen lijden als gevolg van uw deelname aan dit onderzoek. Deze verzekering dekt schade door dood of letsel die het gevolg is van deelname aan het onderzoek, en die zich gedurende de deelname aan het onderzoek openbaart, of binnen vier jaar na beëindiging van de deelname aan het onderzoek. De schade wordt geacht zich te hebben geopenbaard wanneer deze bij de verzekeraar is gemeld.

In geval van schade kunt u zich direct wenden tot de verzekeraar.

De verzekeraar van het onderzoek is:

Naam: Onderlinge Waarborgmaatschappij Centramed b.a.
Adres: Princes Beatrixlaan 35
Postadres: Postbus 90504, 2509 LM ‘s-Gravenhage
Telefoonnummer: 070-5130513

De verzekering biedt een maximum dekking van:

€ 450.000 per proefpersoon en

€ 3.500.000 voor het gehele onderzoek en

€ 5.000.000 per jaar voor alle onderzoeken van dezelfde opdrachtgever.

De dekking van specifieke schades en kosten is verder tot bepaalde bedragen beperkt. Zie voor verdere informatie hieromtrent het Besluit verplichte verzekering bij medisch-wetenschappelijk onderzoek met mensen op de website van de Centrale Commissie Mensgebonden Onderzoek: www.ccmo.nl.

Voor deze verzekering gelden een aantal uitsluitingen. De verzekering dekt niet:

- schade waarvan op grond van de aard van het onderzoek zeker of nagenoeg zeker was dat deze zich zou voordoen;
- schade aan de gezondheid die ook zou zijn ontstaan indien u niet aan het onderzoek had deelgenomen;
- schade die het gevolg is van het niet of niet volledig nakomen van aanwijzingen of instructies;
- schade aan nakomelingen, als gevolg van een nadelige inwerking van het onderzoek op u of uw nakomeling;
- bij onderzoek naar bestaande behandelmethoden: schade die het gevolg is van één van deze behandelmethoden;
- bij onderzoek naar de behandeling van specifieke gezondheidsproblemen: schade die het gevolg is van het niet verbeteren of van het verslechteren van deze gezondheidsproblemen.

Indien u schade heeft geleden door het onderzoek of het vermoeden daarvan heeft dient u zich met de onderzoeker dan wel uw behandelend arts in verbinding te stellen.

Indien bovengenoemde bedragen de schade niet volledig dekken en aangetoond kan worden dat de uitvoering van het onderzoek onzorgvuldig is geweest dan kunt u hiernaast ook het ziekenhuis dat of de industrie die opdracht gegeven heeft tot het onderzoek of het ziekenhuis waar het onderzoek is uitgevoerd aansprakelijk stellen.

Measurements

	anthropometry	osteosonography	Saliva	Blood	Urine
**Day 7 postnatally**	**X**	**X**		**X* +250 μl**	**X* +5 ml**
Laboratory measurements				**IGFBP3**	**LH, FSH, E or A**
**Day 28 postnatally**				**X* +250 μl**	**X 5 ml**
Laboratory measurements				**IGFBP3**	**LH, FSH, E or A**
**Postmenstrual age of 32 weeks**	**X**	**X**			**X 5 ml**
Laboratory measurements					**LH, FSH, E or A**
**0 months corrected age**	**X**	**X**			**X 5 ml**
Laboratory measurements					**LH, FSH, E or A**
**3 months corrected age**	**X**	**X**			**X 5 ml**
Laboratory measurements					**LH, FSH, E or A**
**6 months corrected age**	**X**	**X**	**X**	**X 500 μl**	
Laboratory measurements			**DHEAS**	**IGF-1, IGFBP1, IGFBP3, insulin, glucose, DHEAS**	
**12 months corrected age**	**X**	**X**			
**24 months corrected age**	**X**	**X**	**X**	**X 500 μl**	
Laboratory measurements			**DHEAS cortisol**	**IGF-1, IGFBP1, IGFBP3, insulin, glucose, DHEAS**	
Total volume				1,5 ml	25 ml

GEGEVENS BEPALINGEN ENDOCRINOLOGISCH LABORATORIUM

I

NB. CV % is monster- en concentratieafhankelijk — januari 2007

bepaling eenheid	intra-assay		inter-assay		Bepalingsgrens	methode en fabrikant
	gem.	CV%	gem	CV%	Endo-lab	
**Cortisol**<br>nmol/L	700	3	150<br>500<br>1000	6<br>6<br>8	30	Competitieve immunoassay<br>luminescentie<br>ACS:Centaur<br>Bayer Diagnostics<br>Mijdrecht Nederland
**Cortisol, vrij**<br>nmol/L<br>Speeksel	1.3<br>30	19<br>7	2<br>14<br>30	19<br>7<br>7	1.5	Radio immunoassay<br>gecoate buizen<br>Spectria Orion Diagn.<br>Espoo Finland
**DHEA-Sulfaat**<br>µmol/L<br>Serum en urine	3<br>10	6<br>4	3.0<br>10	10<br>9	0.2	Radio immunoassay<br>Coat-A-Count, DPC<br>Los Angeles USA
**LH**<br>U/L<br>Urine	*5*<br>*40*<br>*75*	*3*<br>*3*<br>*3*	4<br>23	7<br>6	0.1	Immunometrische assay<br>Luminescentie<br>Architect<br>Abbott Laboratories<br>Diagnostics Division<br>Abbott Park, Illinois USA
**FSH**<br>U/L<br>Urine	*5.5*<br>*25*<br>*75*	*3*<br>*3*<br>*3*	5<br>18	6<br>5	0.11	Immunometrische assay<br>Luminescentie<br>Architect<br>Abbott Laboratories<br>Diagnostics Division<br>Abbott Park, Illinois USA
**Insuline**<br>pmol/L	20<br>500<br>1500	4<br>3<br>4	24<br>780<br>3000	8<br>7<br>7		Immunometrische assay<br>Luminescentie<br>ACS:CENTAUR<br>Bayer Diagnostics<br>Mijdrecht Nederland
**Insulin-like Growth Factor-1 (IGF-1)**<br>nmol/L	Hele bereik	5	Hele bereik	5	3.2	Immunometrische assay<br>Luminescentie<br>Immulite 2500<br>DPC<br>Los Angeles<br>USA
**Insulin-like Growth Factor Binding Protein-1**<br>mg/L	4<br>50<br>120	5<br>5<br>5	10<br>100	10<br>6	1,5	Immunoradiometrische assay<br>DSL,<br>Webster Texas<br>USA
**Insulin-like Growth Factor Binding Protein-3**<br>mg/L			Hele bereik	6<br>6	0,2	Immunometrische assay<br>Luminescentie<br>Immulite 2500<br>DPC<br>Los Angeles<br>USA
**Glucose**<br>mmol/l	-	-	4,7<br>18,3	1,5<br>1,5	CCL bepaling	Hexokinase method<br>Roche diagnostics,Mannheim, Germany (Gluco-quant)<br>Modular analytics <P>, Roche diagnostics,Mannheim, Germany
